# Near-field surface plasmon effects on Au-double-slit diffraction for polychromatic light

**DOI:** 10.1186/1556-276X-9-561

**Published:** 2014-10-09

**Authors:** Pin Han

**Affiliations:** 1 Graduate Institute of Precision Engineering, National Chung Hsing University, 250 Kuo Kuang Road, Taichung 402, Taiwan

**Keywords:** Surface plasmon, Au-double-slit, Polychromatic light, Near-field, Spectral switch

## Abstract

The surface plasmon effects on near-field diffraction for polychromatic light are studied. An Au-double-slit is used as the model and Fresnel integral is employed to perform the theoretic analysis. The results are illustrated with numerical examples and they show that, compared with the normal double-slit, the plasmon effect changes the spectral shift from redshift to blueshift and also enhances the intensity peak. This effect can be used in optical data transmission or specific spectral selectors.

## Background

The study of surface plasmons has gained much attention since the discovery of optical transmission enhancement through subwavelength apertures in metal films [1], which can be explained with the excitation of surface plasmons by the incident optical field on a metal-dielectric interface. For a nanostructure metallic double-slit, these plasmon waves travel toward the slits and couple with the field directly transmitted through the slits. In this way, the spectra and even the spatial coherence can be modulated [2]. In the past, the spectral changes induced by normal aperture diffraction have been intensively studied, and an interesting phenomenon called ‘spectral switch’ was discovered [3]. Also, some related applications were suggested, such as lattice spectroscopy [4] or optical data transmission scheme [5]. Recently, the effect of surface plasmons with Au-double-slit for polychromatic light was studied in the far-field [6], which also showed the spectral switch and was controlled by an electro-optic setup. However, in order to enhance the signal intensity and to use this type of optical device in micro/nanoscale, it is worth investigating the plasmonic effects in the near-field (or the so-called Fresnel zone), which is the topic of this work. The results show that the behavior of near-field diffracted spectral intensity with plasmonic effect differs substantially from that without the effect.

## Methods

**Figure 1:**
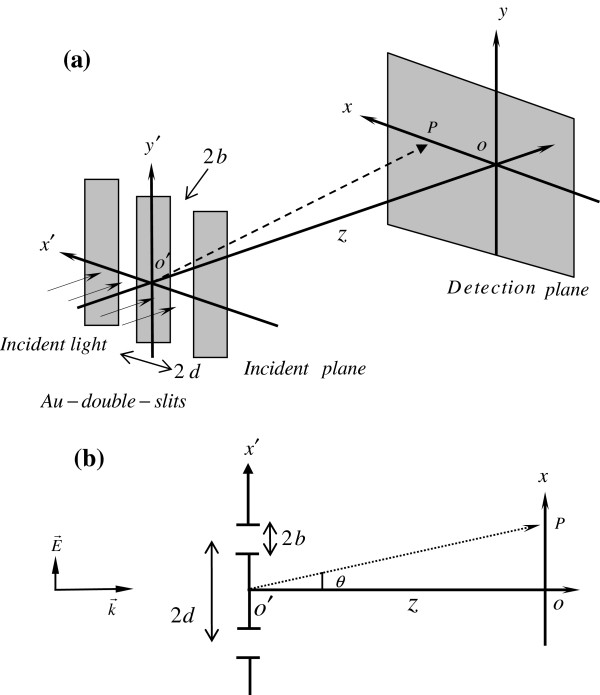
**The Au-double-slit configuration with its corresponding top view.** The Au-double-slit configuration for near-field diffraction. **(a)** The Au-double-slit configuration for near-field diffraction. **(b)** Top view of the configuration in **(a)**.

Consider an Au-double-slit with slit width 2*b* and silt distance 2*d*, as shown in Figure 1a, and a spatially fully coherent polychromatic field incident from the left, which is modeled as a Gaussian profile

(1) $$U^{\prime }\left(\omega \right)=\exp\left\{-{\left[\left(\omega -\omega_{c}\right)\right]}^{2}/2\sigma^{2}\right\}\text{,}$$

where *ω*_*c*_ is the center frequency and *σ* is the bandwidth. To excite surface plasmons along the gold-air interface, TM light polarized in *x*′ polarization as indicated in Figure 1b is used and the field coming out of each slit is

(2) $$U_{Au}^{\prime }\left(\omega \right)=\left[\alpha +\alpha \beta \text{exp}\left(ik_{sp}\cdot 2d\right)\right]\cdot U^{\prime }\left(\omega \right)\text{,}$$

where *α* is the fraction of field directly transmitted, *αβ* is the fraction converted into surface plasmons which travel to the other slit where they reappear as a free propagating field, and *k*_*sp*_ is the surface plasmon polaritons (SPPs) propagation constant. It can be obtained as

(3) $$k_{sp}=\frac{\omega }{c}{\left(\frac{\varepsilon_{m}\cdot \varepsilon_{d}}{\varepsilon_{m}+\varepsilon_{d}}\right)}^{1/2}\text{,}$$

where *ε*_m_ is the dielectric function of gold and *ε*_d_ is that of air. *ε*_m_ can be described with the Drude model as $\varepsilon_{m}=1-\left[\omega_{p}^{2}/\left(\omega^{2}+i\omega \Gamma \right)\right]$, where $\omega_{p}^{2}=1.38\times 10^{16}s^{-1}$ and *Γ* =1.075 × 10^14^ s^-1^ for gold [7,8]. This model fits quite well with the experimental values for wavelength above 650 nm [7]. For fully spatial coherent light with the Au-double-slit, the diffracted light detected at point *P*(*x*,*z*) in the near-field is

(4) $$\begin{array}{ll} \;U_{Au}\left(x,z,\omega \right) & =\frac{\,\exp\left(ikz\right)}{2i}[Fr\left(u_{2}\sqrt{\omega }\right)-Fr\left(u_{1}\sqrt{\omega }\right)\\ & \;+Fr\left(u_{4}\sqrt{\omega }\right)-Fr\left(u_{3}\sqrt{\omega }\right)]\cdot U_{Au}^{\prime }\left(\omega \right)\text{,} \end{array}$$

where *k* = *ω*/*c* is the wavenumber, $Fr\left(u\right)\equiv \int_{0}^{u}\exp\left(i\frac{\pi }{2}\;t^{2}\right)\;dt$ is the Fresnel integral and we set $u_{1}=\sqrt{1/\pi cz}\left(-d-b-x\right)$, $u_{2}=\sqrt{1/\pi cz}\left(-d+b-x\right)$, $u_{3}=\sqrt{1/\pi cz}\left(d-b-x\right)$, and $u_{4}=\sqrt{1/\pi cz}\left(d+b-x\right)$. Note that the Fresnel approximation is used to derive Equation 4 from the Fresnel diffraction integral [9]. For a normal double-slit, Equation 4 can be used by replacing *U*′_*Au*_(*ω*) in Equation 2 with *U*′(*ω*) in Equation 1. The spectral intensity with *I*_*Au*_(*x*,*z*,*ω*) = |*U*_*Au*_(*x*,*z*,*ω*)|^2^ is

(5) $$\begin{array}{ll} \;I_{Au}\left(x,z,\omega \right) & =\left(1/4\right)\left|[Fr\left(u_{2}\sqrt{\omega }\right)-Fr\left(u_{1}\sqrt{\omega }\right)\right)+Fr\left(u_{4}\sqrt{\omega }\right)\\ & \;-Fr\left(u_{3}\sqrt{\omega }\right)]{\left(\left[\alpha +\alpha \beta \text{exp}\left(ik_{sp}\cdot 2d\right)\right]\right|}^{2}\cdot I^{\prime }\left(\omega \right)\\ & \;\equiv M_{Au}\left(x,z,\omega \right)\cdot I^{\prime }\left(\omega \right)\text{,} \end{array}$$

where from Equation 1, *I*′(*ω*) = |*U*′(*ω*)|^2^ = exp{-[(*ω* - *ω*_*c*_)]^2^/*σ*^2^} is the incident spectrum; $M_{Au}\left(x,z,\omega \right)=\left(1/4\right)\left|[Fr\left(u_{2}\sqrt{\omega }\right)-Fr\left(u_{1}\sqrt{\omega }\right)\right)+Fr\left(u_{4}\sqrt{\omega }\right)-Fr\left(u_{3}\sqrt{\omega }\right)]{\left(\left[\alpha +\alpha \beta \text{exp}\left(ik_{sp}\cdot 2d\right)\right]\right|}^{2}\text{,}$ the term in front of *I*′(*ω*) in Equation 5, is named the modifier because it indicates how *I*′(*ω*) is modified to give the diffracted spectrum at *P*. Note that the modifier contains two parts, the double-slit part (the four *Fr*(*u*) terms) and the plasmon part (*α* + *αβ*exp(*ik*_*sp*_ ⋅2*d*)). For normal double-slit, the plasmon part disappears and is replaced by pure apertures; thus, we have

(6) $$\begin{array}{ll} \;I_{Nor}\left(x,z,\omega \right) & =\left(1/4\right)|[Fr\left(u_{2}\sqrt{\omega }\right)-Fr\left(u_{1}\sqrt{\omega }\right)+Fr\left(u_{4}\sqrt{\omega }\right)\\ & \;{-Fr\left(u_{3}\sqrt{\omega }\right)]|}^{2}\cdot I^{\prime }\left(\omega \right)\\ & =M_{Nor}\left(x,z,\omega \right)\cdot I^{\prime }\left(\omega \right)\text{.} \end{array}$$

The subscript Nor denotes the normal double-slit situation. A normal double-slit is a completely opaque configuration except the two open slits, which is usually obtained from an infinitely thin, perfect conducting screen; thus, the field inside the slits is the same as the excitation field and Kirchhoff diffraction integral is applicable. The near-field (Fresnel zone) condition is the following: $N_{c}\theta^{2}\leq 1$, where tan (*θ*) = *x*/*z* is the angle between the line O′P and the optic axis O′O as indicated in Figure 1b and *N*_*c*_ = *x*^2^/*λ*_*c*_*z* is the Fresnel number at center wavelength *λ*_*c*_. Both Equations 5 and 6 are utilized to perform the near-field diffraction calculations; the numerical results and comparisons between the two situations are given in the next section.

## Results and discussion

**Figure 2:**
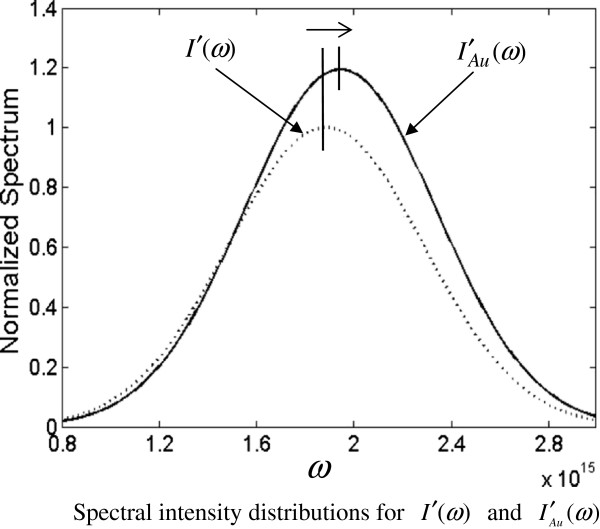
**Spectral intensity distributions for *I*** **′(*ω*) and**$I_{Au}^{\prime }\left(\omega \right)$**.**

**Figure 3:**
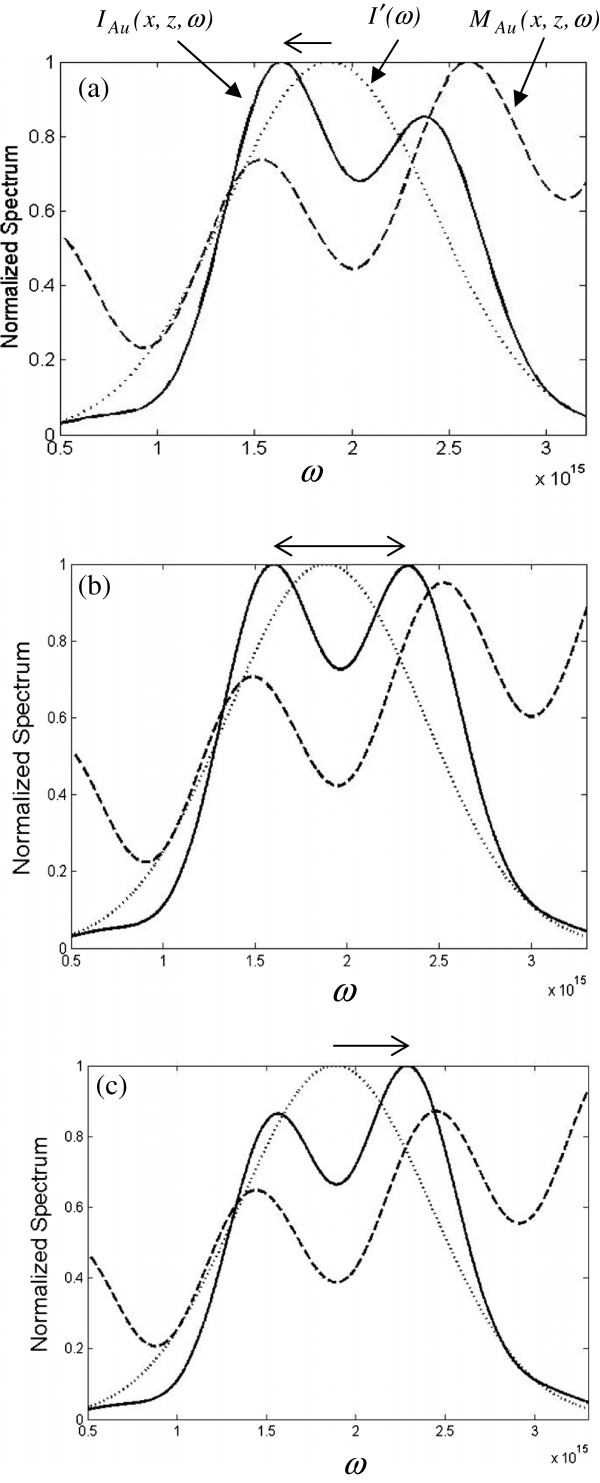
**Spectral intensity distributions for *I***_***Au***_**(*x,z,ω*) (solid line), *M***_***Au***_**(*x,z,ω*) (dashed line), and *I*′(*ω*) (dotted line) for *z *and *x*. *z *=50 um and *x*** **= (a) 13.3, (b) 13.5, and (c) 13.7 um, respectively.**

**Figure 4:**
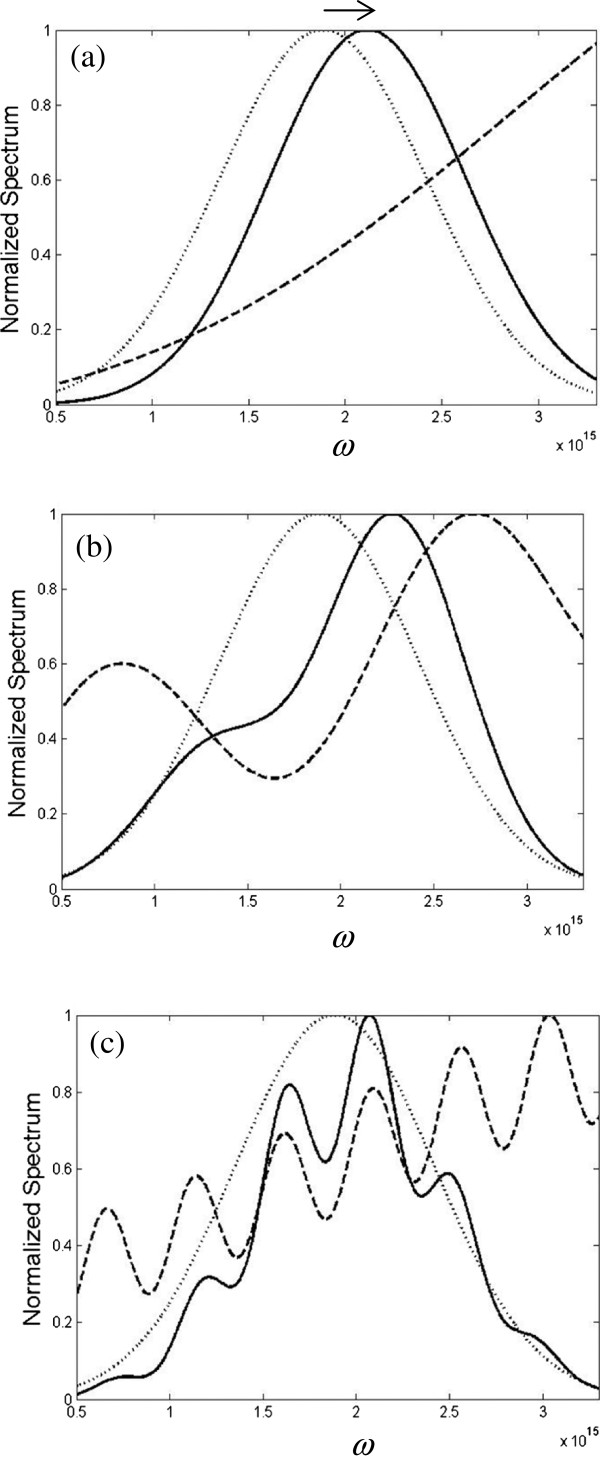
**Spectral intensity distributions for *I***_***Au***_**(*x,z,ω*) (solid line), *M***_***Au***_**(*x,z,ω*) (dashed line), and *I*′(*ω*) (dotted line) for *z *and *x*. *z *=50 um and *x*** **= (a) 0, (b) 10, and (c) 20 um, respectively.**

**Figure 5:**
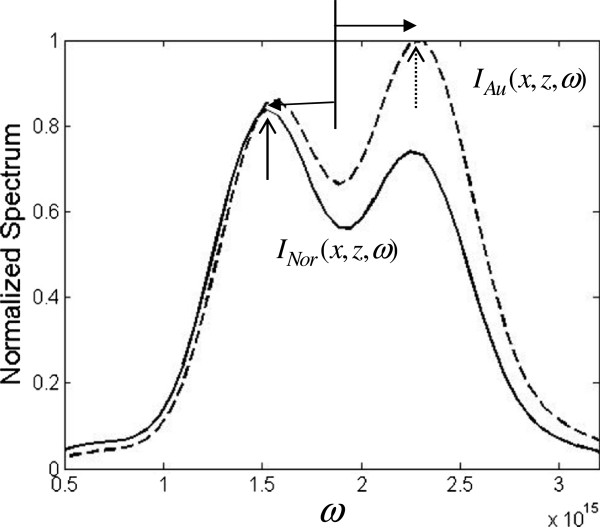
**Spectral intensity distribution for *I***_***Nor***_**( *x, z, ω *) (solid line) and *I***_***Au***_**( *x, z, ω *) (dashed line).**

After the formulation, we can illustrate some features of plasmon effect on double-slit diffraction in the near-field with numerical works. Figure 2 shows the *I*′_*Au*_(*ω*) = |*U*′_*Au*_(*ω*)|^2^ and *I*′(*ω*) = |*U*′(*ω*)|^2^ with Equations 1 and 2 for the following parameters: *ω*_*c*_ =6*π* × 10^14^ s^-1^, *σ* =0.3*ω*_*c*_, 2*b* = *d* =100 nm, thickness =200 nm, *α* =0.99, |*β*| =0.33, arg(*β*) =205^0^[2]; the corresponding center wavelength is *λ*_*c*_ =1,000 nm and wavelength interval is from 770 to 1,430 nm, lying in the interval to which the Drude model for gold is applicable as mentioned above. It is found from Figure 2 that just behind the double-slit the peak intensity of *I*′_*Au*_(*ω*) (solid line) is 1.2 times of that of *I*′(*ω*) (dotted line) because of the surface plasmon enhancement and that the peak frequency is blueshifted with an amount of 0.03*ω*_*c*_, as indicated by the arrow above the figure. In the near-field (Fresnel zone), Figure 3a,b,c shows the behavior of all functions including the incident spectrum *I*′(*ω*) (dotted line), the modifier *M*_*Au*_(*x*,*z*,*ω*) (dashed line), and the diffracted spectrum *I*_*Au*_(*x*,*z*,*ω*) (solid line) for *z* =50 um and *x* =13.3, 13.5, and 13.7 um, respectively. First, checking plot (a), the modifier exhibits an oscillating property that depends on the locations of *x* and influences the incident spectrum and modulates the diffracted spectrum; consequently, there are two peaks in *I*_*Au*_(*x*,*z*,*ω*). It shows that the maximum of the main peak in *I*_*Au*_(*x*,*z*,*ω*) is redshifted (move to lower frequency), as indicated by the arrow above and the amount of the shift is about -0.35*σ*. Then, in plot (b), we see that the two peaks reach the same height when the lateral position varies from 13.3 to 13.5 um. Finally, in plot (c), when the lateral position varies from 13.5 to 13.7 um, the maximum of the main peak is blueshifted (move to higher frequency) and the amount of the shift is about 0.9*σ*. From the three plots, we find that the spectral blue or redshift depends on the lateral detection location and it makes a quick transition at some specific position as shown in plot (b); this discontinuous spectral shift jump is called the spectral switch [10], which has been studied lately and extensively under different situations. To have a better insight about the lateral spatial effect on the spectral behavior, Figure 4a,b,c shows the spectra for *x* =0, 10, 20 um, respectively. It is found that only the blueshift is found because the modifier is monotonously increasing for *x* =0 as shown in Figure 4a. When the value of *x* increases, the oscillating behavior of the modifier becomes more obvious and denser as illustrated in Figure 4b,c, which is reasonable for a Fresnel integral (Cornu spiral) with incremental argument; consequently, the diffraction spectrum is modulated more violently. For the purpose of comparison between the normal and Au double-slit, Figure 5 illustrates the diffracted spectra for *I*_*Nor*_(*x*,*z*,*ω*) (solid line) and *I*_*Au*_(*x*,*z*,*ω*) (dashed line) at *x* =13.7 um and *z* =50 um. The maximum of each function is indicated by solid and dashed vertical arrows. There are two points that are worth mentioning. First, the amplitude of *I*_*Au*_(*x*,*z*,*ω*) is larger than that of *I*_*Nor*_(*x*,*z*,*ω*), and it is about 1.18 times that of normal double-slit. Second, the main peak in Au case is blueshifted, while the main peak in normal case is redshifted as indicated by the horizontal arrows. Thus, the effects of surface plasmon in near-field are twofold: both the amplitude enhancement and large spectral shift change can be obtained. This import result can give us more flexibility to control the polychromatic light through the plasmonic phenomenon in near-field. It is noted that the behavior of the modifier in Equation 5 depends not only on the detection location of *P*(*x*,*z*) but also on the dimension parameters of the slits *b* and *d* because they are coupled in the argument of the Fresnel integral, for example, $u_{1}=\sqrt{1/\pi cz}\;\left(-d-b-x\right)$, and the plasmon phase term exp(*ik*_*sp*_ ⋅2*d*) is related with *d* too. Thus, all these four variables *x*, *z*, *b*, and *d* are coupled and contributed to influence the plasmonic spectral behavior.

## Conclusions

The plasmonic effect on the diffracted spectral behavior of Au-double-slit in the near-field is studied. The analytic formulations for both Au and normal cases are derived by applying Fresnel approximation to the Fresnel diffraction integral. It is found that the incident spectral intensity is enhanced and blueshifted right after the Au-double-slit. Also, the numerical results show that the spectral switch can be found when the lateral positions vary and that the surface plasmons can affect both the magnitude of the diffracted spectral intensity and the spectral shifts, which benefit the control of spectra with plasmons and the potential applications in nano-optic devices.

## Competing interests

The author declares that he has no competing interests.

## Authors' information

Pin Han is now a professor and the head of the Graduate Institute of Precision Engineering, National Chung Hsing University. His main interests of research are optical engineering, optical design, and light wave propagation.
